# Assessment of a Risk-Based Approach for Triaging Mammography Examinations During Periods of Reduced Capacity

**DOI:** 10.1001/jamanetworkopen.2021.1974

**Published:** 2021-03-25

**Authors:** Diana L. Miglioretti, Michael C. S. Bissell, Karla Kerlikowske, Diana S. M. Buist, Steven R. Cummings, Louise M. Henderson, Tracy Onega, Ellen S. O’Meara, Garth H. Rauscher, Brian L. Sprague, Anna N. A. Tosteson, Karen J. Wernli, Janie M. Lee, Christoph I. Lee

**Affiliations:** 1 Division of Biostatistics, Department of Public Health Sciences, University of California Davis School of Medicine, Davis; 2 Kaiser Permanente Washington Health Research Institute, Kaiser Permanente Washington, Seattle; 3 Departments of Medicine and Epidemiology and Biostatistics, University of California, San Francisco; 4 General Internal Medicine Section, Department of Veterans Affairs, University of California, San Francisco; 5 Kaiser Permanente Bernard J. Tyson School of Medicine, Pasadena, California; 6 San Francisco Coordinating Center, California Pacific Medical Center Research Institute, San Francisco; 7 Department of Radiology, University of North Carolina, Chapel Hill; 8 Huntsman Cancer Institute, Department of Population Health Sciences, University of Utah, Salt Lake City; 9 Division of Epidemiology and Biostatistics, University of Illinois at Chicago, Chicago; 10 Office of Health Promotion Research, University of Vermont Cancer Center, Department of Surgery, Larner College of Medicine at the University of Vermont, Burlington; 11 Norris Cotton Cancer Center, The Dartmouth Institute for Health Policy and Clinical Practice, Geisel School of Medicine at Dartmouth, Lebanon, New Hampshire; 12 Department of Radiology, University of Washington School of Medicine, Seattle; 13 Hutchinson Institute for Cancer Outcomes Research, Seattle, Washington; 14 Department of Health Services, University of Washington School of Public Health, Seattle

## Abstract

**Importance:**

Breast cancer screening, surveillance, and diagnostic imaging services were profoundly limited during the initial phase of the coronavirus disease 2019 (COVID-19) pandemic.

**Objective:**

To develop a risk-based strategy for triaging mammograms during periods of decreased capacity.

**Design, Setting, and Participants:**

This population-based cohort study used data collected prospectively from mammography examinations performed in 2014 to 2019 at 92 radiology facilities in the Breast Cancer Surveillance Consortium. Participants included individuals undergoing mammography. Data were analyzed from August 10 to November 3, 2020.

**Exposures:**

Clinical indication for screening, breast symptoms, personal history of breast cancer, age, time since last mammogram/screening interval, family history of breast cancer, breast density, and history of high-risk breast lesion.

**Main Outcomes and Measures:**

Combinations of clinical indication, clinical history, and breast cancer risk factors that subdivided mammograms into risk groups according to their cancer detection rate were identified using classification and regression trees.

**Results:**

The cohort included 898 415 individuals contributing 1 878 924 mammograms (mean [SD] age at mammogram, 58.6 [11.2] years) interpreted by 448 radiologists, with 1 722 820 mammograms in individuals without a personal history of breast cancer and 156 104 mammograms in individuals with a history of breast cancer. Most individuals were aged 50 to 69 years at imaging (1 113 174 mammograms [59.2%]), and 204 305 (11.2%) were Black, 206 087 (11.3%) were Asian or Pacific Islander, 126 677 (7.0%) were Hispanic or Latina, and 40 021 (2.2%) were another race/ethnicity or mixed race/ethnicity. Cancer detection rates varied widely based on clinical indication, breast symptoms, personal history of breast cancer, and age. The 12% of mammograms with very high (89.6 [95% CI, 82.3-97.5] to 122.3 [95% CI, 108.1-138.0] cancers detected per 1000 mammograms) or high (36.1 [95% CI, 33.1-39.3] to 47.5 [95% CI, 42.4-53.3] cancers detected per 1000 mammograms) cancer detection rates accounted for 55% of all detected cancers and included mammograms to evaluate an abnormal mammogram or breast lump in individuals of all ages regardless of breast cancer history, to evaluate breast symptoms other than lump in individuals with a breast cancer history or without a history but aged 60 years or older, and for short-interval follow-up in individuals aged 60 years or older without a breast cancer history. The 44.2% of mammograms with very low cancer detection rates accounted for 13.1% of detected cancers and included annual screening mammograms in individuals aged 50 to 69 years (3.8 [95% CI, 3.5-4.1] cancers detected per 1000 mammograms) and all screening mammograms in individuals younger than 50 years regardless of screening interval (2.8 [95% CI, 2.6-3.1] cancers detected per 1000 mammograms).

**Conclusions and Relevance:**

In this population-based cohort study, clinical indication and individual risk factors were associated with cancer detection and may be useful for prioritizing mammography in times and settings of decreased capacity.

## Key Points

**Question:**

How can imaging facilities optimize the number of breast cancers detected during periods of reduced capacity using clinical indication and individual characteristics?

**Findings:**

In this cohort study including 898 415 individuals with 1 878 924 mammograms, 12% of mammograms with very high and high cancer detection rates accounted for 55% of detected cancers, while 44% of mammograms with very low cancer detection rate accounted for 13% of detected cancers.

**Meaning:**

These findings suggest that triaging individuals most likely to have cancer detected during periods of reduced capacity could result in detecting the most cancers while performing the fewest examinations compared with a non–risk-based approach.

## Teaser

This cohort study assesses strategies to triage patients for mammogram cancer screening during times of reduced capacity.

## Introduction

The coronavirus disease 2019 (COVID-19) pandemic has profoundly impacted the US health care system, including breast cancer screening, surveillance, and diagnostic services.^1,2,3,4^ To rapidly respond to county and state stay-at-home orders during the initial phase of the pandemic, radiology facilities cancelled or reduced nonurgent services, including breast cancer screening and surveillance, and some diagnostic imaging, which allowed health care personnel to shift to pandemic-related efforts and preserve personal protective equipment.^5,6,7,8,9^ The precipitous drop in screening and diagnostic imaging^1,4^ likely reduced the number of breast cancers diagnosed.^10,11^ As COVID-19 case rates continue to increase in waves, radiology facilities face significant scheduling challenges in addressing the backlog of postponed mammograms, reduced staff, and reduced number of mammogram appointment times required to maintain physical distancing and safety protocols, resulting in markedly diminished imaging availability.^12,13^

During periods of reduced capacity or in settings with limited mammography availability, facilities, individuals and their physicians who are determining the urgency of mammography should consider the probability that breast cancer will be detected. Several professional associations have posted guidance for scheduling individuals for breast imaging services during the COVID-19 pandemic based on expert opinion.^13,14,15^ For example, the Society of Breast Imaging^12^ suggests prioritization, from highest to lowest urgency, of patients who need imaging to inform breast cancer surgery, imaging for percutaneous breast biopsy, diagnostic work-up of an abnormal screening examination, short interval follow-up or diagnostic imaging for nonurgent symptoms, and last, screening with possible further prioritization by breast cancer risk. These recommendations do not specifically address how breast cancer risk factors should be considered nor address individuals with a personal history of breast cancer.

We used data representative of the US population from the Breast Cancer Surveillance Consortium (BCSC)^16^ to develop an algorithm that radiology facilities could use to optimize triaging of individuals for mammography examinations during periods of decreased mammography capacity, such as during surge periods of the COVID-19 pandemic.

## Methods

### Study Setting and Cohort

The BCSC registries and the Statistical Coordinating Center received institutional review board approval for active or passive consenting processes or waiver of consent to enroll participants, link and pool data, and perform analysis. All procedures adhered to the Health Insurance Portability and Accountability Act. Registries and the Statistical Coordinating Center received a Federal Certificate of Confidentiality and other protections for the identities of individuals, physicians, and facilities. This report follows the Strengthening the Reporting of Observational Studies in Epidemiology (STROBE) reporting guideline for cohort studies.

Prospective data were collected by 7 BCSC registries^16,17^: Carolina Mammography Registry, Kaiser Permanente Washington, New Hampshire Mammography Network, Vermont Breast Cancer Surveillance System, Sacramento Area Breast Imaging Registry, San Francisco Mammography Registry, and Metropolitan Chicago Breast Cancer Registry. Registries collect individual-level characteristics and clinical information from community radiology facilities^18^ and link to state or regional cancer registries and pathology databases for complete capture of breast cancer diagnoses and benign breast disease.^17^ The study population included 1 878 924 mammograms from 898 415 individuals interpreted by 448 radiologists from 2014 to 2019 across 92 facilities. We excluded mammograms performed within 6 months after a breast cancer diagnosis because they were likely associated with treatment planning.

### Characteristics of Individuals and Mammograms

Age, first-degree family history of breast cancer, breast symptoms, time since last mammogram, and breast cancer history were collected from self-administered questionnaires at the time of mammography or extracted from electronic health records. Self-reported race/ethnicity was categorized as White, Black, Asian or Pacific Islander, Hispanic/Latina, and other or mixed race/ethnicity. Breast cancer history was also obtained by linkage with pathology databases and state or regional cancer registries. Diagnoses of high-risk breast lesions, defined as atypical ductal or lobular hyperplasia or lobular carcinoma in situ, were obtained from pathology databases and cancer registries (for lobular carcinoma in situ).

Clinical indication for the mammogram was assigned by the radiologist or technologist as screening, additional evaluation of a recent mammogram, short-interval follow-up, or diagnostic for clinical signs or symptoms. We further subdivided indications for clinical symptoms into presence of a lump, presence of other symptoms, and unknown. We classified surveillance mammograms (ie, asymptomatic mammograms) among individuals with a breast cancer history using a previously described algorithm with slight modifications.^19^ Mammograms with a nonspecific diagnostic indication (66 382 mammograms [3.5%]) were classified as short-interval follow-up if the most recent prior mammogram was within 3 to 13 months, with a final Breast Imaging Reporting and Data System (BI-RADS)^20^ assessment of 3 (ie, probably benign finding) after all imaging work-up was performed; as an additional evaluation of recent mammogram if the most recent prior mammogram occurred within 3 months and had an initial assessment of 0 (ie, need additional imaging evaluation), 3, 4 (ie, suspicious abnormality), or 5 (ie, highly suggestive of malignant neoplasm), or occurred within 3 to 6 months and had an initial assessment of 0, 4, or 5; or otherwise as a diagnostic mammogram for clinical signs or symptoms. Radiologists categorized BI-RADS breast density^20^ at the time of clinical interpretation as almost entirely fatty, scattered fibroglandular densities, heterogeneously dense, or extremely dense.

### Mammography Outcomes

Breast cancer diagnoses were obtained by linking with pathology databases and state or regional cancer registries.^21^ The final BI-RADS assessment was considered positive if it had an assessment of 4 or 5 and negative if the assessment was 1, 2, or 3.^20^ The cancer detection rate was calculated as number of mammograms with positive final assessment and invasive carcinoma or ductal carcinoma in situ diagnosed within 90 days divided by total number of mammograms.

### Statistical Analysis

We summarized characteristics of the study population by breast cancer history. We estimated cancer detection rate by individual and mammographic characteristics, separately by breast cancer history, and estimated 95% CIs using generalized estimating equations with a working independence correlation structure to account for correlation among mammograms within the same facility.^22^

Classification and regression trees (CART)^23^ were used to identify factors most strongly associated with cancer detection rate. We fit 4 models: screening mammograms in individuals without a breast cancer history, surveillance mammograms in individuals with a breast cancer history, and diagnostic mammograms separately by breast cancer history. All models included age group (<40, 40-49, 50-59, 60-69, ≥70 years), time since last mammogram (first mammogram, 1 year, 2 years, ≥3 years), first-degree family history of breast cancer, and breast density as potential predictors. Models for diagnostic mammograms also included clinical indication as additional evaluation of a recent mammogram, short-interval follow-up, or clinical signs or symptoms subdivided by lump, other symptoms, and unknown symptoms. Models for individuals without a breast cancer history also included history of a high-risk lesion. Models for individuals with a breast cancer history also included years since diagnosis (<5, 5 to <10, ≥10, unknown).

We set a maximum tree depth (ie, number of levels in the decision tree) of 10 and a maximum number of leaves (ie, final subgroups) of 10. We required at least 10% of the sample in each leaf. The maximum tree depth achieved was 5, the maximum number of leaves was 7, and pruning was not required. To internally validate the model, we split the study population into 2 random samples, fit the CART models to each sample, and compared the results to the overall model; we found no clinically meaningful differences.

We grouped the final subgroups from the 4 models into 5 risk groups: very high (>50), high (22-50), moderate (10-22), low (5-10), and very low (<5) cancer detection rate per 1000 mammograms. Thresholds were chosen based on the observed cancer detection rates and clinical expertise. Given limitations of CART in identifying small subgroups with clinically important risk differences, we separated screening examinations of individuals with a history of a high-risk lesion into their own subgroup, because of their relatively high cancer detection rate. We plotted the cumulative percentage of cancers detected by the cumulative percentage of mammograms according to the cancer detection rates for the subgroups selected by the CART models, sorted from high to low. We created simplified flowcharts that facilities can use for scheduling under 2 scenarios of reduced capacity.

Statistical analyses used SAS statistical software version 9.4 software (SAS Institute). Data were analyzed from August 10 to November 3, 2020.

## Results

Table 1 shows the characteristics of 898 415 individuals contributing 1 878 924 mammograms, including 1 722 820 mammograms in individuals without a breast cancer history and 156 104 mammograms in individuals with a breast cancer history. Most mammograms were performed for screening among individuals without a breast cancer history (1 465 854 mammograms [85.1%]) or surveillance among individuals with a breast cancer history (139 337 mammograms [89.3%]). Most mammograms were performed in individuals aged 50 to 69 years (1 113 174 mammograms [59.2%]; mean [SD] age, 58.6 [11.2] years); and 204 305 (11.2%) were Black, 206 087 (11.3%) were Asian or Pacific Islander, 126 677 (7.0%) were Hispanic or Latina, and 40 021 (2.2%) were another race/ethnicity or mixed race/ethnicity.

**Table 1.** Characteristics of Mammograms by Personal History of Breast Cancer

Characteristic	Personal history of breast cancer, No. (%)^a^	
	No (n = 1 722 820)	Yes (n = 156 104)
Clinical indication		
Screening	1 465 854 (85.1)	NA
Surveillance	NA	139 337 (89.3)
Additional evaluation	119 995 (7.0)	5189 (3.3)
Short interval follow-up	41 455 (2.4)	6904 (4.4)
Evaluation of a breast problem		
Lump	23 739 (1.4)	2011 (1.3)
Other symptoms	22 568 (1.3)	1833 (1.2)
Symptoms unknown	49 209 (2.9)	830 (0.5)
Time since last mammogram, y		
First mammogram	97 577 (6.0)	812 (0.5)
<1	166 835 (10.2)	31 638 (20.6)
1	870 730 (53.3)	108 085 (70.4)
2	302 054 (18.5)	8692 (5.7)
≥3	196 076 (12.0)	4288 (2.8)
Missing	89 548 (5.2)	2589 (1.7)
Age, y		
<40	37 626 (2.2)	1242 (0.8)
40-49	387 927 (22.5)	10 069 (6.5)
50-59	538 138 (31.2)	32 987 (21.1)
60-69	488 596 (28.4)	53 453 (34.2)
≥70	270 533 (15.7)	58 353 (37.4)
Time since breast cancer diagnosis, y		
<5	NA	55 102 (39.6)
5-10	NA	36 860 (26.5)
≥10	NA	47 183 (33.9)
Missing	NA	16 959 (10.9)
Race/ethnicity		
White	1 130 661 (67.9)	112 663 (73.1)
Black	188 949 (11.3)	15 356 (10.0)
Asian or Pacific Islander	190 534 (11.4)	15 553 (10.1)
Hispanic/Latina	119 207 (7.2)	7470 (4.8)
Other or mixed	36 922 (2.2)	3099 (2.0)
Missing	56 547 (3.3)	1963 (1.3)
First degree family history of breast cancer		
No	1 324 684 (82.0)	111 363 (74.1)
Yes	290 993 (18.0)	39 019 (25.9)
Missing	107 143 (6.2)	5722 (3.7)
BI-RADS breast density		
Almost entirely fatty	157 386 (9.3)	11 857 (7.8)
Scattered fibroglandular densities	737 261 (43.8)	75 265 (49.4)
Heterogeneously dense	659 054 (39.1)	57 191 (37.6)
Extremely dense	130 941 (7.8)	7921 (5.2)
Missing	38 178 (2.2)	3870 (2.5)
History of high-risk lesion^b^		
No	1 714 009 (99.5)	140 045 (89.7)
Yes	8811 (0.5)	16 059 (10.3)

Abbreviations: BI-RADS, Breast Imaging Reporting and Data System; NA, not applicable.

^a^ Percentages are given as column percentage among nonmissing, except for the missing category, which are given as the column percentage missing of total.

^b^ Includes atypical hyperplasia or lobular carcinoma in situ.

Table 2 shows the cancer detection rates by breast cancer history, clinical indication, and individual characteristics. The overall cancer detection rate was 11.5 (95% CI, 10.7-12.4) cancers per 1000 mammograms. Among individuals without a breast cancer history, the cancer detection rate was 10.9 (95% CI, 10.2-11.6) cancers per 1000 mammograms overall and ranged from 5.0 (95% CI, 4.7-5.4) cancers per 1000 screening mammograms to 65.0 (95% CI, 57.5-73.4) cancers per 1000 mammograms for diagnostic evaluation of a breast lump. The cancer detection rate was 6.9 (95% CI, 6.1-8.0) cancers per 1000 mammograms for first screening mammograms and increased with screening interval, from 3.9 (95% CI, 3.7-4.2) cancers per 1000 mammograms for annual screens to 8.1 (95% CI, 7.5-8.7) cancers per 1000 mammograms for screens when it had been 3 or more years since the individual’s last mammogram. The cancer detection rate for screening mammograms increased from 2.8 (95% CI, 2.6-3.1) cancers per 1000 mammograms in individuals aged 40 to 49 years to 8.0 (95% CI, 7.4-8.7) cancers per 1000 mammograms in individuals 70 years or older. The cancer detection rate was 12.7 (95% CI, 10.1-16.0) cancers per 1000 mammograms in individuals with a history of high-risk lesion.

**Table 2.** Cancer Detection Rate per 1000 Mammograms in Individuals With and Without a Personal History of Breast Cancer

Characteristic	Personal history of breast cancer, cancer detection rate per 1000 mammograms (95% CI)	
	No (n = 1 722 820)	Yes (n = 156 104)
Overall^a^	10.9 (10.2-11.6)	18.6 (16.9-20.5)
Clinical indication		
Diagnostic mammograms	44.1 (40.9-47.5)	67.2 (53.3-84.3)
Additional evaluation	53.0 (48.6-57.6)	138.2 (123.6-154)
Short interval follow-up	11.9 (10.6-13.4)	10.0 (6.5-15.3)
Evaluation of a breast problem		
Lump	65.0 (57.5-73.4)	98.0 (77.7-122.8)
Other symptoms	49.2 (42.4-57.0)	81.9 (59.5-111.8)
Symptoms unknown	23.2 (20.4-26.5)	40.9 (29.7-56.0)
Screening/surveillance mammograms^b^	5.0 (4.7-5.4)	12.7 (11.3-14.3)
Time since last mammogram, y		
First mammogram	6.9 (6.1-8.0)	NA
<1	NA	15.1 (10.3-22.2)
1	3.9 (3.7-4.2)	10.8 (9.9-11.9)
2	5.3 (5.0-5.7)	18.6 (15.7-22.0)
≥3	8.1 (7.5-8.7)	36.8 (29.9-45.2)
Age, y		
<40	3.0 (2.2-4.3)	9.2 (5.1-16.7)
40-49	2.8 (2.6-3.1)	9.3 (7.3-12.0)
50-59	4.1 (3.9-4.5)	10.3 (8.8-11.9)
60-69	6.0 (5.6-6.4)	11.6 (10.5-12.9)
≥70	8.0 (7.4-8.7)	15.8 (13.7-18.2)
Time since breast cancer diagnosis, y		
<5	NA	7.7 (6.8-8.8)
5-10	NA	10.4 (8.5-12.6)
≥10	NA	17.5 (15.2-20.2)
First degree family history of breast cancer		
No	4.6 (4.3-4.9)	11.9 (10.6-13.4)
Yes	6.8 (6.4-7.2)	15.0 (12.8-17.5)
BI-RADS breast density		
Almost entirely fatty	4.0 (3.3-4.8)	12.0 (9.0-15.9)
Scattered fibroglandular densities	5.1 (4.8-5.5)	12.4 (11.0-13.9)
Heterogeneously dense	5.3 (4.9-5.7)	13.3 (11.5-15.4)
Extremely dense	4.4 (3.9-5.0)	10.0 (7.8-12.9)
History of high-risk lesion^c^		
No	5.0 (4.7-5.3)	NA
Yes	12.7 (10.1-16.0)	NA

Abbreviations: BI-RADS, Breast Imaging Reporting and Data System; NA, not applicable.

^a^ The overall cancer detection rate for screening, surveillance and diagnostic mammograms was 11.5 (95% CI, 10.7-12.4) per 1000 mammograms.

^b^ Screening mammograms among women without a personal history of breast cancer, surveillance mammograms among women with a personal history of breast cancer.

^c^ Includes atypical hyperplasia or lobular carcinoma in situ.

Among individuals with a personal history of breast cancer, the cancer detection rate was 18.6 (95% CI, 16.9-20.5) cancers per 1000 mammograms overall and varied by clinical indication, from 10.0 (95% CI, 6.5-15.3) cancers per 1000 short-interval follow-up mammograms to 138 (95% CI, 123.6-154) cancers per 1000 mammograms for additional evaluation of a recent abnormal mammogram. The cancer detection rate for surveillance mammograms generally increased with increasing time since last mammogram, from 10.8 (95% CI, 9.9-11.9) cancers per 1000 mammograms for 1 year to 36.8 (95% CI, 29.9-45.2) cancers per 1000 mammograms among individuals whose last mammogram had been 3 or more years ago, except that mammograms performed less than 1 year since last mammogram had a cancer detection rate of 15.1 (95% CI, 10.3-22.2) cancers per 1000 mammograms. The cancer detection rate for surveillance mammograms increased with age, from 9.2 (95% CI, 5.1-16.7) cancers per 1000 mammograms in individuals younger than 40 years to 15.8 (95% CI, 13.7-18.2) cancers per 1000 mammograms in individuals 70 years or older. The cancer detection rate for surveillance mammograms increased with time since diagnosis, from 7.7 (95% CI, 6.8-8.8) cancers per 1000 mammograms in individuals diagnosed within 5 years to 17.5 (95% CI, 15.2-20.2) cancers per 1000 mammograms for individuals diagnosed 10 or more years prior.

The 12.0% of mammograms with very high (89.6 [95% CI, 82.3-97.5] to 122.3 [95% CI, 108.1-138.0] cancers detected per 1000 mammograms) or high (36.1 [95% CI, 33.1-39.3] to 47.5 [95% CI, 42.4-53.3] cancers detected per 1000 mammograms) cancer detection rates accounted for 55.0% of all detected cancers (Table 3). The Figure shows the cumulative percentage of cancers detected by the cumulative percentage of mammograms according to subgroup cancer detection rates, sorted from highest to lowest. The 3.1% of mammograms classified as very high risk of cancer detection accounted for 25.3% of detected cancers. Adding the 8.9% of mammograms at high risk of cancer detection (29.7% of total cancers), for a total of 12.0% of mammograms, accounted for a total of 55.0% of detected cancers. Adding the 7.2% of mammograms at moderate risk, for a total of 19.2% of mammograms, accounted for a total of 64.2% of all detected cancers. Adding the 36.7% of mammograms with low risk, for a total of 55.8% of all mammograms, accounted for a total of 86.9% of all detected cancers. The 44.2% of mammograms with very low risk of cancer detection accounted for the remaining 13.1% of all detected cancers.

**Table 3.** Percentage of Mammograms, Percentage of Detected Cancers, and Cancer Detection Rate by Risk Group Characterized by Clinical Indication, Personal History of Breast Cancer, Time Since Diagnosis, and Age

Risk group (% of mammograms) [% of detected cancers]	Clinical indication	PHBC and time since diagnosis	Age, y	Mammograms, No. (%) (n = 1 878 924)	Detected cancers, No. (%) (n = 21 624)	Cancer detection rate per 1000 mammograms (95% CI)
Very high risk (3.1) [25.3]	Evaluation for abnormal mammogram or lump^a^	PHBC, any	Any	8030 (0.4)	982 (4.5)	122.3 (108.1-138.0)
	Evaluation for abnormal mammogram or lump	No PHBC	≥60	50 199 (2.7)	4499 (20.8)	89.6 (82.3-97.5)
High risk (8.9) [29.7]	Short-interval follow-up or evaluation for symptoms other than lump^a^	No PHBC	≥60	35 085 (1.9)	1668 (7.7)	47.5 (42.4-53.3)
	Evaluation for symptoms other than lump	PHBC, any	Any	1833 (0.1)	75 (0.3)	40.9 (29.7-56.0)
	Evaluation for abnormal mammogram or lump^a^	No PHBC	<60	129 539 (6.9)	4676 (21.6)	36.1 (33.1-39.3)
Moderate risk (7.2) [9.1]	Surveillance	PHBC, ≥10 y^b^	Any	58 762 (3.1)	1063 (4.9)	18.1 (15.9-20.6)
	Short-interval follow-up	PHBC, any	≥70	2271 (0.1)	35 (0.2)	15.4 (10.0-23.7)
	Screen, women with history of high-risk lesion	No PHBC	Any	6381 (0.3)	81 (0.4)	12.7 (10.1-16.0)
	Short-interval follow-up or evaluation for symptoms other than lump	No PHBC	≤60	42 143 (2.2)	493 (2.3)	11.7 (9.9-13.9)
	Surveillance	PHBC, <10 y	≥70	26 181 (1.4)	303 (1.4)	11.6 (9.8-13.6)
Low risk (36.7) [22.7]	Surveillance	PHBC, <10 y	<70	54 394 (2.9)	410 (1.9)	7.5 (6.5-8.7)
	Screen, first or >1 y since last mammogram^c^	No PHBC	≥50	465 689 (24.8)	3466 (16.0)	7.4 (6.9-8.0)
	Short-interval follow-up	PHBC, any	<70	4633 (0.2)	34 (0.2)	7.3 (4.4-12.1)
	Screen, annual	No PHBC	≥70	163 922 (8.7)	1002 (4.6)	6.1 (5.6-6.7)
Very low risk (44.2) [13.1]	Screen, annual	No PHBC	50-69	504 484 (26.8)	1915 (8.9)	3.8 (3.5-4.1)
	Screen, any	No PHBC	<50	325 378 (17.3)	922 (4.3)	2.8 (2.6-3.1)

Abbreviation: PHBC, personal history of breast cancer.

^a^ Includes evaluation of clinical signs or symptoms, but symptoms are not specified.

^b^ Includes unknown time since breast cancer diagnosis.

^c^ Includes unknown time since last mammogram.

**Figure.**
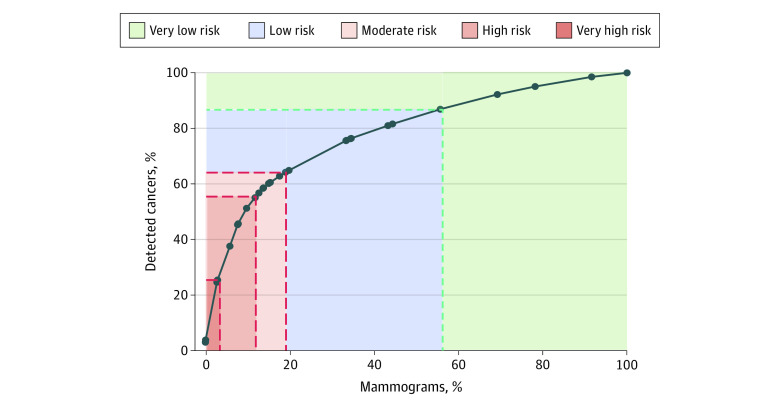
Cumulative Percentage of Detected Cancers by Cumulative Percentage of Mammograms According to Cancer Detection Rate From 29 Subgroups Selected by the Classification and Regression Tree Models, Sorted From High to Low Black dots indicate cumulative percentage of detected cancers by cumulative percentage of mammograms.

Table 3 summarizes the risk groups, ordered from highest to lowest cancer detection rate; expanded descriptions of the subgroups selected by the CART models are in the eTable in the Supplement. The CART models identified clinical indication, age, and time since diagnosis for individuals with breast cancer history as the characteristics most strongly associated with cancer detection rate, with time since last mammogram important for the low and very low risk groups. BI-RADS density and family history were selected for some models, but differences by these 2 risk factors were not clinically meaningful, and subgroups were combined into the same risk groups. The cancer detection rate was considered very high or high for clinical indications of additional evaluation of a recent mammogram or diagnostic work-up of a breast lump in all individuals, diagnostic work-up of symptoms other than lump in individuals with a breast cancer history, and short-interval follow-up or diagnostic work-up of symptoms other than lump in individuals aged 60 years or older without a breast cancer history (Table 3). The cancer detection rate was considered moderate for surveillance mammograms in individuals diagnosed with breast cancer 10 or more years prior, short-interval follow-up examinations in individuals aged 70 years or older with a breast cancer history, screening examinations in individuals with a history of a high-risk breast lesion, short-interval follow-up or diagnostic work-up of symptoms other than lump in individuals younger than 60 years, and surveillance examinations in individuals less than 10 years since diagnosis. The cancer detection rate was considered low for surveillance mammograms in individuals with a breast cancer history diagnosed less than 10 years prior, first screening mammograms and screens performed more than 1 year since last mammogram in individuals aged 50 years or younger, short-interval follow-up examinations in individuals younger than 70 years with a personal history of breast cancer, and annual screens in individuals 70 years or older. The cancer detection rate was considered very low for annual screens in individuals aged 50 to 69 years (3.8 [95% CI, 3.5-4.1] cancers detected per 1000 mammograms) and all screens in individuals younger than 50 years (2.8 [95% CI, 2.6-3.1] cancers detected per 1000 mammograms) (Table 3).

The eFigure in the Supplement provides examples of simple algorithms that facilities could use to triage mammograms if operating at approximately 12% capacity, which would allow scheduling mammograms with high and very high cancer detection rate, (Table 3), or 20% capacity, which would allow scheduling mammograms with moderate and higher cancer detection rates. Facilities could modify the algorithms to better reflect the services they provide. Mammograms that meet the criteria in the flowcharts should be scheduled as soon as possible; otherwise, the mammograms can be scheduled as capacity allows.

## Discussion

Using data from 92 US-based radiology facilities in the BCSC, this cohort study identified subgroups of individuals undergoing mammograms with combinations of clinical indications and individual characteristics associated with high, moderate, and low cancer detection rates. Radiology facilities could use this risk stratification model to triage individuals for mammography during periods of reduced capacity, such as that which occurred during the start of the COVID-19 pandemic. Clinicians could use our results to counsel individuals about how urgently they should seek breast imaging based on their breast symptoms, breast cancer history, age, and time since last mammogram. We demonstrate that triaging individuals at highest risk of having cancer detected could result in detecting the most cancers while performing the fewest examinations compared with a non–risk-based approach. For example, a non–risk-based approach resulted in 11.5 cancers detected per 1000 mammograms (corresponding to the overall cancer detection rate in our study) while a risk-based approach limiting to the 12.1% of mammograms with high or very high risk of cancer detection detected 55.0% of cancers and resulted in at least a 3- to 10-fold greater cancer detection rate (36-122 cancers per 1000 mammograms). In contrast, the cancer detection rate for the 44.2% of mammograms with the lowest risk was 3.8 cancers or less per 1000 and accounted for 13.1% of detected cancers. The low cancer detection rate in these individuals should be considered, along with patient preferences, when deciding about the safety of postponing imaging owing to limited capacity, such as during pandemic surges when individuals may also experience risks and anxiety about contracting an infectious disease.

The American College of Radiology posted a “Return to Mammography Care” toolkit,^24^ encompassing general resources for facilities, including pamphlets and letter templates to reassure patients regarding COVID-19 safety measures. Our results and triaging flowcharts complement this resource by providing direct, detailed evidence that supports risk-based scheduling of individuals most likely to have a cancer detected based on recent data from more than 1.8 million mammograms interpreted by more than 400 radiologists from more than 90 imaging facilities. In contrast to Society of Breast Imaging guidance based on expert opinion,^12^ our data-driven analysis suggests a different triage order for certain groups. For instance, while the Society of Breast Imaging recommends prioritizing short-interval follow-up examinations over screening examinations, we found that woman with breast cancer diagnosed more than 10 years prior undergoing surveillance mammography and individuals with a history of high-risk lesions undergoing screening had higher cancer detection rates than most individuals requiring short-interval follow-up.

Infrastructure should be developed and resources should be allocated to facilitate risk-based algorithm implementation. Challenges include population-based risk factor ascertainment to triage those already scheduled, and software for real-time implementation when individuals call to schedule their examinations. Electronic health records could be adapted to automate prioritization, given they typically contain risk factor information. As part of our ongoing project, we are developing scripts that schedulers can use to encourage individuals with the highest risk to receive care as soon as possible despite the pandemic, and to reassure individuals whose care may be deferred owing to their low risk.

While our analysis was motivated by limited mammography capacity at the start of the COVID-19 pandemic, our results are applicable to other situations and clinical settings. For example, several medical centers have experienced cyberattacks that limited access to electronic health records.^25,26^ Some centers had to greatly reduce clinical care until records were back online. The strategy of offering and promoting medical services based on risk is relevant for other cancer screening tests, including cervical cancer screening, low-dose computed tomography lung cancer screening, prostate cancer screening, and colonoscopy.^27,28,29,30,31,32^ Additionally, our approach may be applicable to other diseases, such as hypertension and diabetes, for which visits to clinicians may be limited by the pandemic or in regions where health care services and clinicians are in short supply. Using data-driven strategies to identify subgroups at highest risk of disease during times of limited capacity may lead to less downstream strain on an already taxed health care system. When capacity is limited, health care systems must ensure that the patients with the highest risk receive care.

### Limitations

Our study has several limitations. Some facilities may only offer screening or diagnostic mammography or may not collect all the risk factors in our models. These facilities could remove nonapplicable subgroups or combine subgroups. Small subgroups may not have been identified by the CART models. We were unable to assess individuals at greater than 20% lifetime risk owing to a strong family history of cancer, genetic variation, or chest radiation before age 30 years. Our algorithms do not consider individual preferences or worries about breast cancer or about contracting COVID-19. We used cancer detection as a measure of potential benefit of mammography; however, benefits of detecting breast cancer may depend on other factors such as life expectancy (eg, age and comorbidities at detection) and characteristics of the cancer detected (eg, tumor subtype).

## Conclusions

This cohort study found that in situations of reduced clinical capacity, risk-based triaging of mammograms based on clinical indication, breast symptoms, breast cancer history, and age could maximize cancer detection. We found that the 12% of mammograms with the highest cancer detection rates accounted for 55% of detected cancers. The approach we propose may provide a model for prioritizing use of screening, diagnostic, or other medical services for other types of conditions during periods of reduced capacity or in resource-limited settings.

## Notes

Supplement. **eTable.** Description of Subgroups From Classification and Regression Tree Models **eFigure.** Flowcharts Showing Which Mammograms Should be Scheduled as Soon as Possible for Facilities Operating at Limited Capacity
