# Supplementary material for: Assessment of a Risk-Based Approach for Triaging Mammography Examinations During Periods of Reduced Capacity
Source: JAMA Netw Open. 2021 Mar 25;4(3):e211974. doi: 10.1001/jamanetworkopen.2021.1974 (PMC7994953; doi:10.1001/jamanetworkopen.2021.1974)
Supplement: Supplement. — eTable. Description of Subgroups From Classification and Regression Tree Models eFigure. Flowcharts Showing Which Mammograms Should be Scheduled as Soon as Possible for Facilities Operating at Limited Capacity [file jamanetwopen-e211974-s001.pdf]

Supplemental Online Content

Miglioretti DL, Bissell MCS, Kerlikowske K, et al. Assessment of a risk-based approach for triaging mammography examinations during periods of reduced capacity. *JAMA Netw Open*. 2021;4(3):e211974. doi:10.1001/jamanetworkopen.2021.1974

eTable. Description of Subgroups From Classification and Regression Tree Models

eFigure. Flowcharts Showing Which Mammograms Should be Scheduled as Soon as Possible for Facilities Operating at Limited Capacity

This supplemental material has been provided by the authors to give readers additional information about their work.

© 2021 Miglioretti DL et al. *JAMA Network Open.*

eTable. Description of Subgroups From Classification and Regression Tree Models

Risk group (% of mammograms, % of detected cancers)	Clinical indication and relevant risk factors	History of breast cancer and time since diagnosis, y	Age, y	Mammograms (N=1,878,924) %	Mammograms Cumulative %	Cancers (N=21,624) %	Cancers Cumulative %	Cancer Detection Rate per 1000 (95% CI)
Very high risk (3.1%, 25.3%)	Evaluation for abnormal mammogram or lump[a]	PHBC, ≥10 y	Any	0.2%	0.2%	2.9%	2.9%	178.5 (153.0, 207.2)
	Evaluation for abnormal mammogram or lump[a]	5-9 years	Any	0.1%	0.3%	0.8%	3.7%	102.3 (87.5, 119.3)
	Evaluation for abnormal mammogram or lump[a]	No PHBC	≥60	2.7%	3.0%	20.8%	24.6%	89.6 (82.3, 97.5)
	Evaluation for abnormal mammogram or lump[a]	<5 years	Any	0.1%	3.1%	0.8%	25.3%	62.5 (52.6, 74.2)
High Risk (8.9%, 29.7%)	Evaluation for abnormal mammogram or lump[a]	No PHBC	50-59	2.9%	6.0%	12.2%	37.5%	48.5 (44.5, 52.9)
	SIFU or evaluation for symptoms other than lump[a]	No PHBC	≥60	1.9%	7.9%	7.7%	45.3%	47.5 (42.4, 53.3)
	Evaluation for symptoms other than lump	PHBC, any	Any	0.1%	8.0%	0.3%	45.6%	40.9 (29.7, 56.0)
	Evaluation for lump[a]	No PHBC	<50	2.0%	9.9%	5.6%	51.2%	32.4 (28.7, 36.6)
	Evaluation for abnormal mammogram	No PHBC	<50	2.0%	12.0%	3.8%	55.0%	21.9 (19.2, 25.0)
Moderate Risk (7.2%, 9.1%)	Surveillance in women with family history	PHBC, ≥10 y[b]	Any	0.9%	12.9%	1.7%	56.7%	21.6 (18.4, 25.3)
	Surveillance in women without a family history	PHBC, ≥10 y[b]	≥70	1.0%	13.9%	1.6%	58.4%	19.1 (16.2, 22.6)
	SIFU	PHBC, any	70	0.1%	14.0%	0.2%	58.5%	15.4 (10.0, 23.7)
	Surveillance in women without a family history	PHBC, ≥10 y[b]	<70	1.2%	15.2%	1.6%	60.1%	14.7 (12.8, 16.9)
	Screening women with history of high-risk lesion	No PHBC	Any	0.3%	15.5%	0.4%	60.5%	12.7 (10.1, 16.0)
	SIFU or evaluation for symptoms other than lump	No PHBC	<60	2.2%	17.8%	2.3%	62.8%	11.7 (9.9, 13.9)
	Surveillance	PHBC, <10 y	≥70	1.4%	19.2%	1.4%	64.2%	11.6 (9.8, 13.6)
Low Risk (36.7%, 22.7%)	Surveillance, >1 year since last mammogram	PHBC, <10 y	<70	0.8%	20.0%	0.7%	64.9%	9.9 (7.8, 12.5)
	First screen or >1 year since last mammogram[c]	No PHBC	≥60	13.5%	33.5%	10.7%	75.6%	9.1 (8.5, 9.8)
	SIFU in women with not dense breasts	PHBC, any	<70	0.1%	33.6%	0.1%	75.7%	8.1 (4.8, 13.7)
	Annual surveillance	PHBC, 5-9 y	<70	1.0%	34.6%	0.6%	76.3%	7.4 (5.9, 9.2)
	SIFU in women with dense breasts	PHBC, any	<70	0.1%	34.7%	0.1%	76.4%	6.5 (3.7, 11.6)
	Annual screen	No PHBC	≥70	8.7%	43.4%	4.6%	81.0%	6.1 (5.6, 6.7)
	Surveillance	PHBC, <5 y	<70	1.1%	44.5%	0.6%	81.6%	6.0 (5.0, 7.1)
	First screen or >1 year since last mammogram[c]	No PHBC	50-59	11.3%	55.8%	5.3%	86.9%	5.4 (4.9, 5.9)
Very Low Risk (44.2%, 13.1%)	Annual screen	No PHBC	60-69	13.5%	69.3%	5.3%	92.2%	4.6 (4.2, 5.0)
	First screen or >1 year since last mammogram[c]	No PHBC	<50	8.9%	78.3%	2.8%	95.1%	3.6 (3.2, 4.1)
	Annual screen	No PHBC	50-59	13.4%	91.6%	3.5%	98.6%	3.0 (2.8, 3.3)
	Annual screen	No PHBC	<50	8.4%	100.0%	1.4%	100.0%	2.0 (1.8, 2.2)

Abbreviations: PHBC = personal history of breast cancer, y = years, SIFU = short interval follow up, CI=confidence interval

© 2021 Miglioretti DL et al. *JAMA Network Open.*

[a]Includes evaluation of clinical signs or symptoms, but symptoms are not specified.
[b]Includes unknown time since breast cancer diagnosis.
[c]Includes unknown time since last mammogram.

© 2021 Miglioretti DL et al. *JAMA Network Open.*

eFigure. Flowcharts Showing Which Mammograms Should be Scheduled as Soon as Possible for Facilities Operating at Limited Capacity
(a) 12% and (b) 20% operating capacity. All other mammograms should be scheduled as capacity allows.

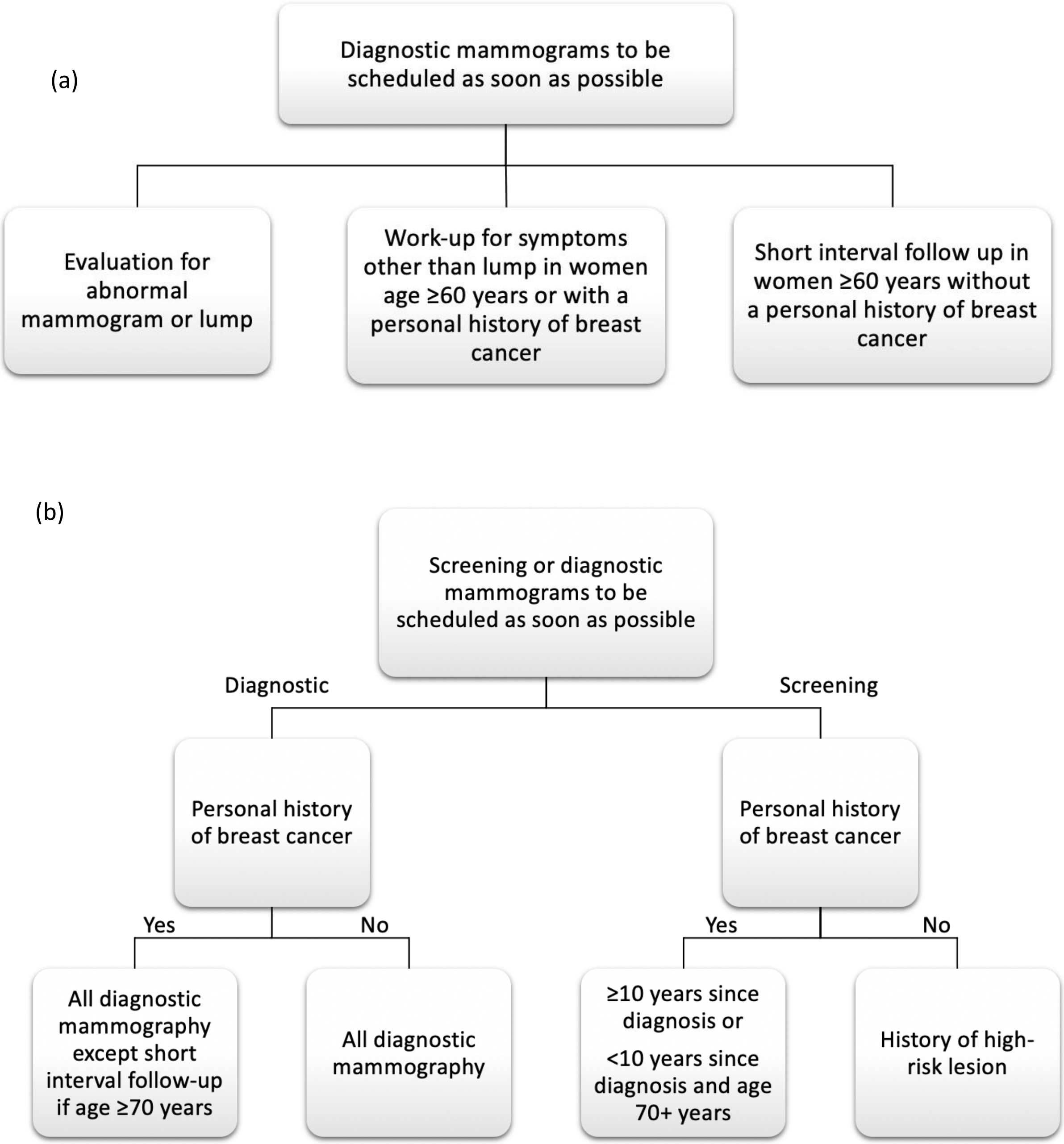

© 2021 Miglioretti DL et al. *JAMA Network Open.*
